# Tinnitus: The Sound of Stress?

**DOI:** 10.2174/1745017901814010264

**Published:** 2018-10-31

**Authors:** Patricia Ciminelli, Sergio Machado, Manoela Palmeira, Mauro Giovanni Carta, Sarah Cristina Beirith, Michelle Levitan Nigri, Marco André Mezzasalma, Antonio Egidio Nardi

**Affiliations:** 1 Laboratory of Panic & Respiration, Institute of Psychiatry, Federal University of Rio de Janeiro, Rua Venceslau Bras, 71 CIPE. Botafogo, Rio de Janeiro, Brazil; 2 Lagoa Federal Hospital, Ministry of Health, Rio de Janeiro, Brazil; 3 Physical Activity Neuroscience, Physical Activity Sciences Postgraduate Program, Salgado de Oliveira University, Niterói, Brazil; 4 Universitá degli studi di Cagliari, Cagliari, Italy

**Keywords:** Tinnitus, Psychosocial stress, Psychosocial comorbidities, Annoyance, Stress, Psychological

## Abstract

**Background:**

Emotional stress is frequently associated with otologic symptoms as tinnitus and dizziness. Stress can contribute to the beginning or worsening of tinnitus.

**Objective:**

The objective of the study is to evaluate the presence of stress symptoms in patients with chronic, subjective tinnitus, and correlate its presence to annoyance associated with tinnitus.

**Methods:**

This is a cross-sectional study. One hundred and eighty patients with chronic, subjective tinnitus were included. Patients answered the Tinnitus Handicap Inventory (THI) to evaluate the impact of tinnitus in the quality of life and answered the Lipp's inventory symptoms of stress for adults (ISSL). The data obtained was organized using Excel® 2010, mean values, linear regression and p-value were calculated.

**Results:**

Of the 180 patients included in the study, 117 (65%) had stress symptoms, 52 of the 117 (44%) were in the resistance phase and 23 of the 117 (20%) in the exhaustion phase, the remaining was in the alert phase. There was a clear progressive increase in stress as THI raised, with more impact of tinnitus in quality of life.

**Conclusion:**

The presence of stress symptoms, measured by ISSL was observed in most of our patients with chronic subjective tinnitus, specially in the resistance and exhaustion phases and it is directly associated with tinnitus annoyance.

## 1 INTRODUCTION

Stress is defined as the result of psychological and physical conditions that threaten the normal functioning of ones body [1]. It is frequently related to numerous medical conditions, including tinnitus. Stress is associated with events difficult to control or manage, related to social, physical, economic, emotional or occupational demands [2].

Tinnitus is a very common auditory disorder affecting approximately 10-15% of the population. Although tinnitus is commonly caused by auditory system damages, the role of emotional and psychological factors inducing and maintaining annoyance has been proven in recent tinnitus studies [3].

Stress is many times attributed as a cause of tinnitus and we often see in the clinic that patients complain that their tinnitus gets worse after stressful situations. The evidence that stress is related to tinnitus is based on studies that show high psychiatric comorbidity related to the symptom. About 10– 60% of chronic tinnitus patients suffer from depressive disorders and 28–45% present with clinically relevant anxiety symptoms [4, 5]. It has been frequently observed that many tinnitus patients present with psychological or psychiatric distress before or during the onset and evolution of tinnitus [6]. Some studies that have used stress questionnaires in tinnitus patients found a high prevalence of self-reported levels of stress. Gomaa *et al.,* found that among 100 patients with tinnitus only 25 didn´t have stress; 44 had mild to moderate stress and 31 severe to extreme levels of stress. There is probably a cause and effect relationship between tinnitus and stress [7].

The aim of the present study is to evaluate the presence of stress symptoms in patients with chronic, subjective tinnitus, using the Lipp's inventory of symptoms of stress for adults (ISSL), as well as to correlate its presence to the annoyance associated to tinnitus, applying the Tinnitus Handicap Inventory (THI). The hypothesis suggested in this cross-sectional study is that higher THI scores are correlated with worst stress levels.

## 2 METHODS

One hundred and eighty patients with chronic (for more than 6 months), subjective tinnitus, with ages 27-79 years were evaluated at a tinnitus clinic in the otorhynolaryngology department at a referral hospital in our city, according to an established clinical protocol for tinnitus patients. Before agreeing to participate in the study, patients signed the informed consent. The study was approved by the local ethic committee, approval number 42441514.0.0000.5291. All patients, who were referred to our clinic from January 2014 to January 2015, were submitted to routine exams for tinnitus diagnosis and were included in the study. Patients answered the THI and the visual analog scale, to evaluate the impact of tinnitus in the quality of life and answered the ISSL, to evaluate the presence of stress symptoms, which is composed by 37 somatic items and 19 psychological items. These items are organized in three phases of stress: alert, resistance and exhaustion phases. They also answered the Mini International Neuropsychiatric Interview (MINI) Questionnaire v. 5.0 for the investigation of the presence of concomitant psychiatric disorders.

The inclusion criteria were:

- Presence of tinnitus for at least 6 months.
- Ability to answer the questionnaires.
- Patient agreement to participate in this study.

The exclusion criteria were:

- Mild and Severe cognitive impairment.
- Diagnosis of central nervous system tumors, including cerebellum-pontine angle (CPA) tumors.
- Recent surgery or otologic procedure, including implantable hearing devices.
- Presence of psychiatric disorders diagnosed by the MINI.

### 2.1 Statistical Analysis

The THI and the ISSL scores in the analysis are reported with mean and standard deviation.

The level of significance used as a reference was set at p ≤ 0.05.

Inferential statistics were performed using Pearson's correlation performed with the Statistical Package for the Social Sciences 23.0 (SPSS).

## 3 RESULTS

One hundred and eighty patients were included in the study, 75 (41,6%) male and 105 (58,4%) female. The age ranged from 27 to 79 years, mean 59.86 years (SD 12.5). The time since the beginning of tinnitus was: from 6 months to 4 years in 93 patients (51,6%), from 5 to 10 years in 60 (33,3%) and more than 10 years in 27 (15%) patients. None of the patients had psychiatric disorders nor were taking medications targeting the central nervous system or ototoxic medications.

**Fig. (1):**
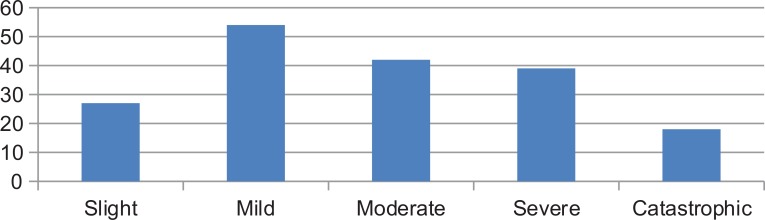
THI scores.

The mean THI score was 43.43 (SD 23.99) and the mean visual analog scale score was 6.91 (SD 14.95). Grade I or slight tinnitus in THI was found in 27 (15%) patients, grade II or mild, in 54 (30%) patients, grade III or moderate, in 42 (23,3%) patients, grade IV or severe, in 39 (21,6%) patients and grade V or catastrophic, in 18 (10%) patients (Fig. **1**).

**Fig. (2):**
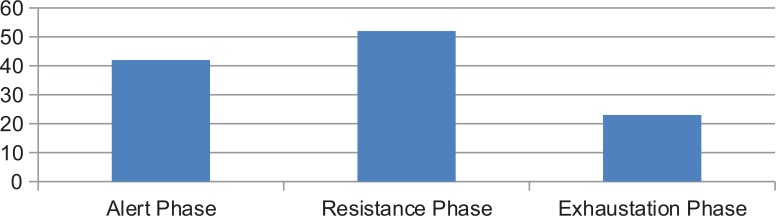
Stress phases in tinnitus patients with stress.

Of the 180 patients included in the study, 117 (65%) had stress symptoms, 52 (44%) of the 117 were in the resistance phase and 23 of 117 (20%) in the exhaustion phase, the remaining patients were in the alert phase of stress (Fig. **2**).

In the slight tinnitus group, 12 (44.4%) patients had stress symptoms. In the mild tinnitus group of patients, 30 (55.5%) had stress. Thirty-three (78,5%) of the patients with grade III tinnitus had stress symptoms; Twenty four (61.5%) in grade IV and all patients (100%) in grade V had stress. We noticed a clear progressive increase in stress as THI gets higher, with more impact in quality of life. The prevalence in each group is represented in Fig. (**3**).

To evaluate the proposed hypothesis (H1), a linear regression with stress being the dependent variable and THI the independent value was done. The *p*-value found was 0.00576, demonstrating that the higher the annoyance, the more stressed patients are.

## 4 DISCUSSION

The results presented show a high occurrence of stress symptoms in tinnitus patients evaluated in a reference tinnitus clinic, specially in the more advanced phases, the resistance and exhaustion phases. We observed a clear increase in stress symptoms in patients with higher THI scores, being stress present in all patients with catastrophic tinnitus. To our knowledge, this is the first study in Brazil using a stress scale in tinnitus patients.

There is enough evidence supporting the understanding that tinnitus induces stress. However, still little is known about stress being responsible for the appearance or worsening of tinnitus. It has been frequently observed that many tinnitus patients present with psychological or psychiatric distress before or during the onset and evolution of tinnitus [8]. Which comes first is still unknown but authors agree that stress is obviously related to tinnitus and directly associated to its annoyance.

One of the earliest published studies showing an evidence between the onset of tinnitus to psycho-social distress was made by John Harrison Curtis, describing two of five patients that related the beginning of tinnitus to a psycho-social stress caused by death of a close family member [9]. One hundred and seventy years later, two large studies published important epidemiological information concerning the association of psychosocial stress with tinnitus [9, 10]. The first study demonstrated that the probability of developing tinnitus is approximately the same for highly stressed persons as it is for persons exposed to occupational noise [9], stress seems as important as auditory damage for causing tinnitus. Importantly, the authors also have noticed that psycho-social stress contributes to worsening of tinnitus symptoms. The studies describe that exposure to high levels of stress and occupational noise, together, doubles the probability of developing tinnitus. In the second study, approximately one-third of the working population observed and complained of hearing problems, tinnitus or both. Tinnitus and the duration and magnitude of stress were linearly associated [10]. Both studies included more than 10,000 subjects each, which provides great statistical strength relating tinnitus to stress. Mazurek *et al.,* [6, 11], described higher scores in the “worries” and “tension” subscales of the Perceived Stress Questionnaire in patients with disturbing chronic tinnitus when compared to those with non-disturbing tinnitus. Similarly, in our study, 65% of our tinnitus patients had stress symptoms, measured by the Lipp's Stress Symptoms Scale.

For many patients, with increased reactivity to stress, tinnitus may work as an alarm signal, at least at its onset, informing the patient that something could be wrong or that something potentially dangerous could be happening and stress factors are clearly related to this reaction. Aspects concerning this danger alarm and the way patients react to and face the symptom can explain the epidemiological differences between the incidence of referred tinnitus (10–15%) and that of disabling tinnitus (2%). In other words, tinnitus becomes a disabling symptom in subjects chronically exposed to stress who are unable to switch off the alarm signal or to neutralize the effect of the stressors. In other individuals, the same factors or diseases could provoke tinnitus without inducing annoyance or distress because these subjects are skilled to cope with tinnitus as a stressor, with a real capacity to restore normal body stability. Individual capacity to neutralize stress factors is strictly specific for each subject: the progression from alarm to exhaustion stress phases is specific for each patient. In a referral tinnitus clinic, one would expect a higher number of patients in resistance and exhaustion phases, as found in our study. It is demanding to identified the stress signals during the alarm phase to prevent a progression toward the resistance phase and, especially, exhaustion phase. These later phases can lead to chronic disabling tinnitus, in which there are an important emotional-affective activation [12, 13].

Assuming that tinnitus patients have a greater amount of stress reactivity, maladjustment to daily stress situations could be a consequence. It has been demonstrated that decompensated tinnitus patients use more maladaptive coping strategies compared to controls [14-17]. Concerning the physiological factors, maladaptive stress reactivity in chronic tinnitus patients should lead to excessive reactivity in the autonomous nervous system. Some studies addressed and evidenced the role of abnormal physiological stress reactions in the onset and maintenance of tinnitus symptoms [18-20]. The high efficacy of psychotherapy in the treatment of tinnitus sufferers argues in favor of stress reactivity related to tinnitus distress [21], considering that most of these treatment programs address coping strategies to stress. Also, studies testing the effect of relaxation training emphasize psychophysiological factors in chronic tinnitus patients [22, 23]. Many patients may also benefit from the use of drugs targeting the central nervous system in order to reduce anxiety and stress associated symptoms [24]

The information collected here, indirectly imply the requirement for psychological assessment during the diagnosis of tinnitus patients. Psychological intervention with a goal of stress-management strategies appears to be an indispensable element in tinnitus treatment, especially important to use in very early stages of tinnitus, before the chronification of plastic changes has taken place, considering that stress is highly associated to tinnitus, as a cause or effect.

Here, we discuss a limitation of our study, the lack of a control group. Our intention was solely to describe the presence of stress and to stratify its stages and correlate with tinnitus annoyance in a selected group of patients referred to a specialized tinnitus clinic.

## CONCLUSION

The presence of stress symptoms, measured by the Lipp's Adult Stress Tinnitus Inventory (ISSL), was observed in the majority of our patients with chronic subjective tinnitus, specially in the resistance and exhaustion phases and it seems to be directly associated to tinnitus annoyance. There’s a close relationship between psychological disorders, including stress, and the occurrence and maintenance of tinnitus, causing an impact on patient's quality of life. This article motivates professionals to consider stress an important symptom to be diagnosed and treated in tinnitus patients.

## Figures and Tables

**Fig. (3):**
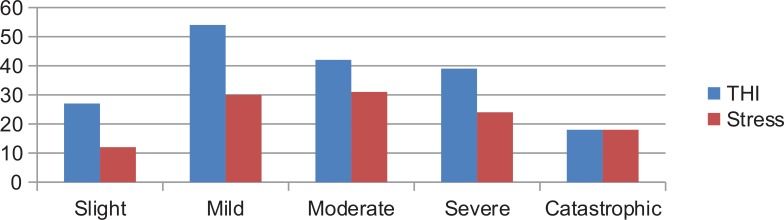
Number of patients with stress in each THI grade.
